# Current approaches to flexible intramedullary nailing for bone lengthening in children

**DOI:** 10.1007/s11832-016-0781-1

**Published:** 2016-11-08

**Authors:** Dmitry Popkov, Pierre Lascombes, Pierre Journeau, Arnold Popkov

**Affiliations:** 1 Russian Ilizarov Scientific Centre for Restorative Traumatology and Orthopaedics, 6 M. Ulyanova Street, Kurgan, 640014 Russian Federation; 2 Division of Paediatric Orthopaedics, Hôpitaux Universitaires de Genève, rue Willy Donzé 6, 1211 Geneva 14, Switzerland; 3 CHU Brabois, Hôpital d’Enfants, Chirurgie Infantile Orthopédique, Rue du Morvan, 54500 Vandoeuvre, France

**Keywords:** Lengthening, Ilizarov technique, Flexible intramedullary nailing, Hydroxyapatite-coated intramedullary nails

## Abstract

Limb-length discrepancies and extremity deformities are among the most common non-traumatic orthopaedic conditions for which children are hospitalised. There is a need to develop new treatment options for lower-limb length discrepancy in order to ameliorate treatment outcomes, avoid or reduce rates of complication and provide early rehabilitation. The authors report on the basic principles, experimental and clinical data, advantages, problems and complications of a combined technique associating the Ilizarov method and flexible intramedullary nailing (FIN) in limb lengthening and deformity correction in children. They describe features of the use of hydroxyapatite-coated intramedullary nails in patients with certain metabolic bone disorders and in cases where bone consolidation has been compromised. The advantages of bone lengthening using a combined technique (circular fixator plus FIN) are a lower healing index, quicker distraction-consolidation, a reduced rate of septic and bone complications, the ability to correct deformities gradually and the increased stability of bone fragments during the external fixation period and after frame removal.

## Introduction

Limb-length discrepancies and extremity deformities are among the most common non-traumatic orthopaedic conditions for which children are referred to hospital [1]. New treatment options developed for lower-limb length discrepancies include “guided growth”, hexapod external fixators, lengthening over an intramedullary nail and lengthening then nailing [2–5]. These techniques have expanded the indications for the surgical management of limb-length discrepancies and decreased the incidence and severity of complications in bone lengthening. Leg lengthening using external fixators has now become an accepted, well-established procedure based on the principles of the Ilizarov method, where distraction osteogenesis encourages the spontaneous consolidation of lengthened and regenerated bone [6, 7].

The incidence of fixator-related problems, however, can prolong the duration of external fixation [8, 9]. Patients must often wear fixators for long periods and the healing index (HI) varies from 35 to 58 days/cm, depending on the publication [5, 10, 11]. Longer periods of fixator attachment are associated with numerous complications: local and deep infections, joint stiffness, delayed consolidation, axial deviation and fractures after frame removal, and a significant psychological impact on the patient [12–15]. Lengthening over nail in adults or fully implantable lengthening nails have been described as techniques which permit the early removal of external fixators or make them unnecessary, providing protection against refracture and earlier rehabilitation [3, 16]. However, in children, the use of rigid intramedullary nails alone can cause physeal injuries and proximal femoral osteonecrosis [17]. Major complications have been reported, including osteomyelitis, mechanical failure of the intramedullary device, prolonged bone consolidation, transfusion requirements, collapse of the lengthened segment with breakage of locking screws and the risk of the nail becoming unreachable inside the canal [18–20]. Furthermore, the presence of an open physis meant that lengthening over nail or implantable nails are contraindicated in children, and an implantable nail can only be used after epiphyseal closure [21].

In paediatric limb-lengthening, approaches for the prophylactic stabilisation of lengthened femurs in children include Rush^®^ pins, unreamed interlocking nails [22] or flexible intramedullary nailing (FIN) [23]—“lengthening then rodding”. However, when using the FIN technique, manipulating the bone during frame removal or nail implant can lead to fractures. In 101 femoral lengthening operations, Schiedel et al. observed five fractures occurring in connection with fixator removal and then seven more in connection with in situ FIN [22]. Another disadvantage of the technique is the risk of infection due to the one-stage change from the external to the internal fixation.

The FIN technique involves bone fixation by means of two bent elastic nails inserted so that they curve in two opposite directions. It was initially described for the management of paediatric fractures, and its efficacy has been proven [24, 25]. In order to reduce the external fixation period in paediatric limb-lengthening and to avoid any risks related to the one-stage change from the external to the internal procedure, we have combined these limb-lengthening methods: progressive distraction with a circular external fixator and FIN applied during the same procedure.

The present article reports on the basic principles, experimental and clinical data, advantages, problems and complications of a combined technique associating the Ilizarov method and FIN. The external fixator can be a classic Ilizarov fixator or any kind of modern external fixator.

### Experimental data [26]

In an experimental study conducted in 28 dogs, the results of tibia lengthening using the conventional Ilizarov technique (group I, 14 animals) were compared with the results of tibia lengthening done using an Ilizarov device and FIN (group II, 14 animals). In group II, the FIN was made up of two 1.5 mm diameter stainless-steel nails with opposing curves positioned in the same plane. The distraction period was 28 days for both groups.

**Fig. 1:**
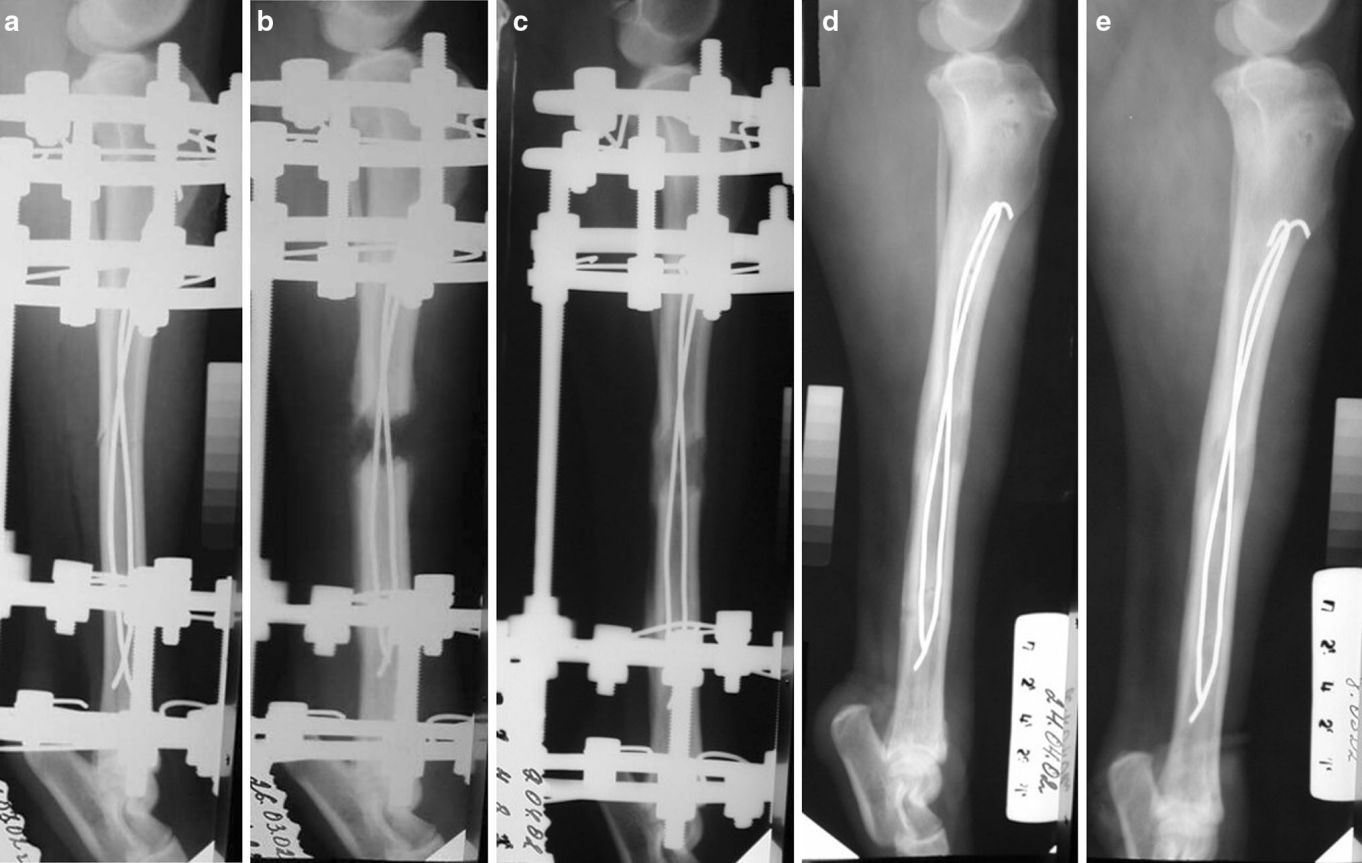
Radiographs of right tibia (dog). Tibia lengthening using an Ilizarov fixator plus FIN: **a** Ilizarov fixator and intramedullary nails in place, osteotomy of tibia and fibula; **b** X-ray image taken after 28-day lengthening (end of distraction period); **c** complete bone consolidation achieved after 14-day fixation period; **d** day of external fixator removal; **e** 32 days after frame removal, no deformities or fractures in lengthened tibia

Radiological images of group II showed more intense and extensive bone regeneration (Fig. 1), which forced an increase in the distraction speed for some dogs. After 2 weeks, the regeneration was well structured and completely filled the interfragmentary gap. The regenerated area was wider than the tibia. After about 15 days of fixation, the regenerated area was completely consolidated in all the animals. Neither fracture nor deformity occurred after removal of the external fixator because the regenerated area was already solid, and also because the dog’s bones were protected by the FIN.

Comparatively, bone consolidation in group I (conventional Ilizarov technique) had only been achieved in half of the animals in the 30–60 days of the fixation period.

**Fig. 2:**
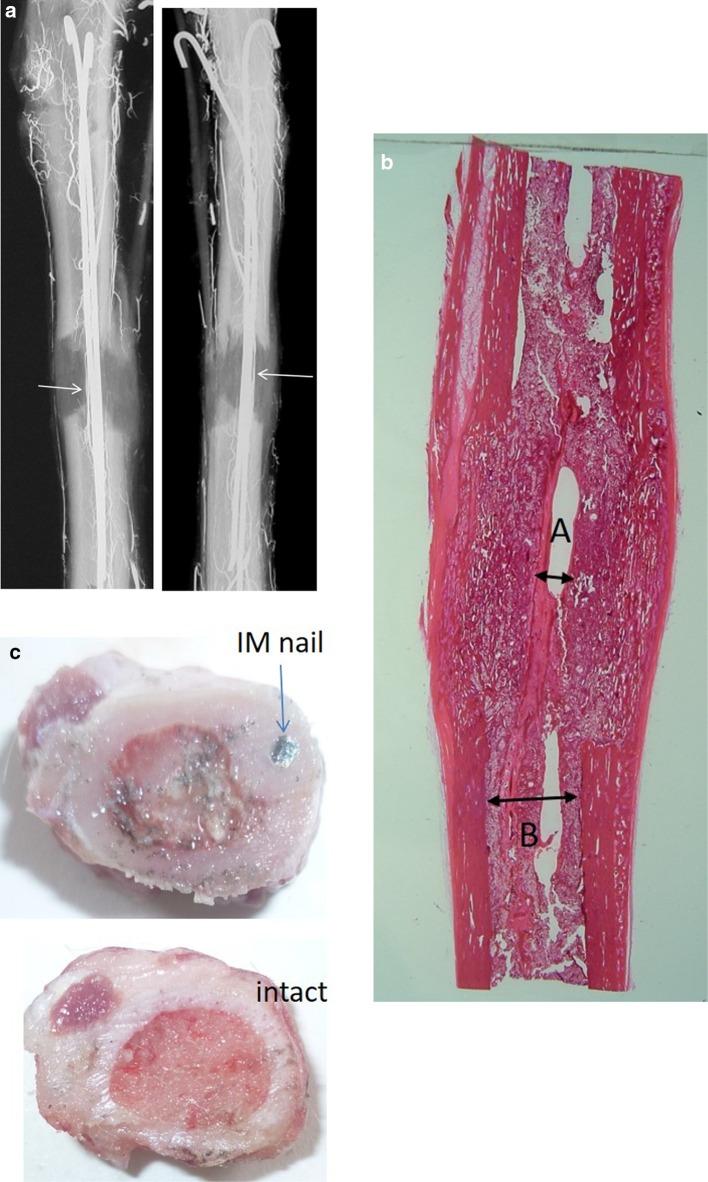
Morphological study: **a** medullary artery was still visible at the end of the distraction period; **b** disappearance of fibrous “growth zone” layer of the regenerated bone and presence of a cortical continuity by day 15 of the fixation period, extensive bone trabeculae observed along alignment of the intramedullary nails, and the diameter of the conglomerate “nail plus surrounding ossification” structure filled 40% of the medullary canal; **c** intramedullary nail surrounded by newly formed bone tissue, thickened cortices (*above*) in comparison to intact healthy tibia (*below*)

Arteriographies were performed at the beginning of lengthening, at the end of the fixation period and after removing the frame and nails. The medullary artery was visible in group I’s arteriographies but also in those of group II’s dogs of group II (Fig. 2a).

By day 15 of the fixation period, histology (Fig. 2b) showed the disappearance of the fibrous “growth zone” layer of the regenerated bone, and the cortical continuity in all the group II dogs, but this was not the case in group I. Indeed, complete bone union was observed 2 weeks after lengthening had been stopped. Furthermore, extensive bone trabeculae were observed along the length of the intramedullary nails (Fig. 2c).

An experimental study suggested that an intramedullary elastic-nail diameter of 20–25% of the medullary canal was the most appropriate with which to avoid any wires and half-pins blocking the external fixator and to allow the nail to glide smoothly along the inner wall of medullary canal during limb lengthening. Indeed, the diameter of the combined structure (conglomerate) of the “nail plus surrounding ossification” had expanded to 35–40% of medullary canal by the end of fixation period (Fig. 2c). In paediatric traumatology, however, it is well known that the diameter of flexible intramedullary nails must be at least 40% of that of the medullary canal in order to obtain stable fixation [27]. Finally, the optimal geometric parameters for intramedullary nails were determined in a biomechanical study modelling the stresses and strains exerted on a bent intramedullary nail inserted into the medullary canal [28].

Thus, experimental and biomechanical studies have proven that the combined method, using a circular external fixator and FIN, in no way contradicts the principles of the Ilizarov method for bone lengthening: elasticity and stability of fixation; preserved intramedullary circulation which stimulates endosteal and periosteal bone formation; and the ability to maintain an optimal rate of gradual lengthening or deformity correction.

### Features of surgical technique

FIN for limb lengthening in hospitals should comply with several basic rules. Depending on the degree of lengthening selected, skin incisions and entry holes should be made more or less close to the osteotomy site. Different approaches have been described in the Nancy University manual [24]. For bifocal humeral or femoral lengthening, however, it is preferable to make two holes—one distal and one proximal—into the cortex, with two nails in a mixed antegrade and retrograde arrangement, and each nail passing through both osteotomies.

**Fig. 3:**
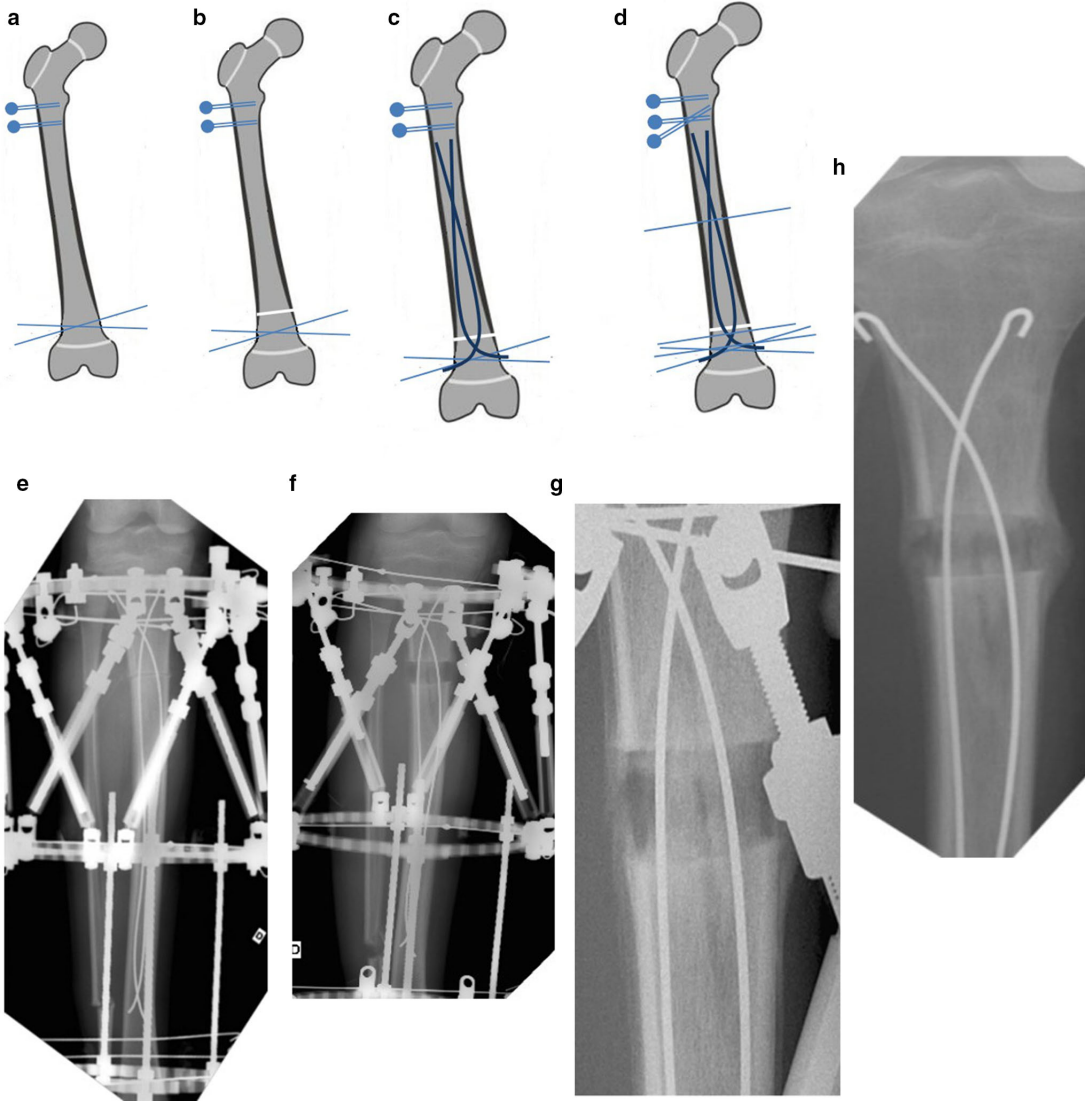
Procedural steps: **a** application of external fixator with only two proximal half-pins (or wires) and two distal wires; **b** percutaneous osteotomy; **c** insertion of intramedullary nails; **d** external osteosynthesis is completed by adding some wires and half-pins connected to the proximal, medial, and distal rings and arches; **e** X-ray image of tibia at beginning of lengthening–combined osteosynthesis with FIN and Taylor Spatial Frame^®^; **f** X-ray image of tibia on day 20 of distraction period; **g** X-ray image of tibia on day 25 of fixation period, bone consolidation; **h** X-ray image after frame removal

The procedure itself consists of several steps (Fig. 3a–d). For example, for femoral monofocal lengthening, the initial steps are standard: an external fixator is applied and then a percutaneous osteotomy is performed. However, to begin with, only two wires are inserted into the distal ring and two wires or half pins into the proximal arch or ring. This facilitates the insertion of two nails. Two incisions are made at the metaphysis, close to the osteotomy site. Two entry holes are created using an awl at an oblique angle to the osteotomy, within 10–20 mm of the growth plate. Rotating the curved tip allows the nail to avoid the external fixator wires inside the femur. Nails must be carefully moved forward, one at a time, as far as the osteotomy site, pushed through the site, and then directed towards the opposite metaphysis. The trailing ends of the nails are then bent more than 90° to prevent any internal migration during the distraction. They are subsequently trimmed, leaving ~5–10 mm above the bone surface, and the skin is closed. As the last step, external osteosynthesis is completed by adding some wires and half-pins to the external fixator’s proximal, middle and distal rings and arcs.

Because the nails have to be inserted through the metaphysis nearest to the osteotomy, they should be bent so that their apex is located at the regeneration site by the end of the distraction period. For example, while tibia lengthening is in progress, the apexes of antegrade intramedullary nails gradually move and finally reach the regenerated area by the end of the distraction period (Fig. 3e–h). Based on past results of limb lengthening, we suggest using nails with a diameter of 20–25% of the medullary canal and bent up to 60° in order to obtain stable bone fragments. A nail curve over 60° may lead to them blocking the canal during the distraction.

**Fig. 4:**
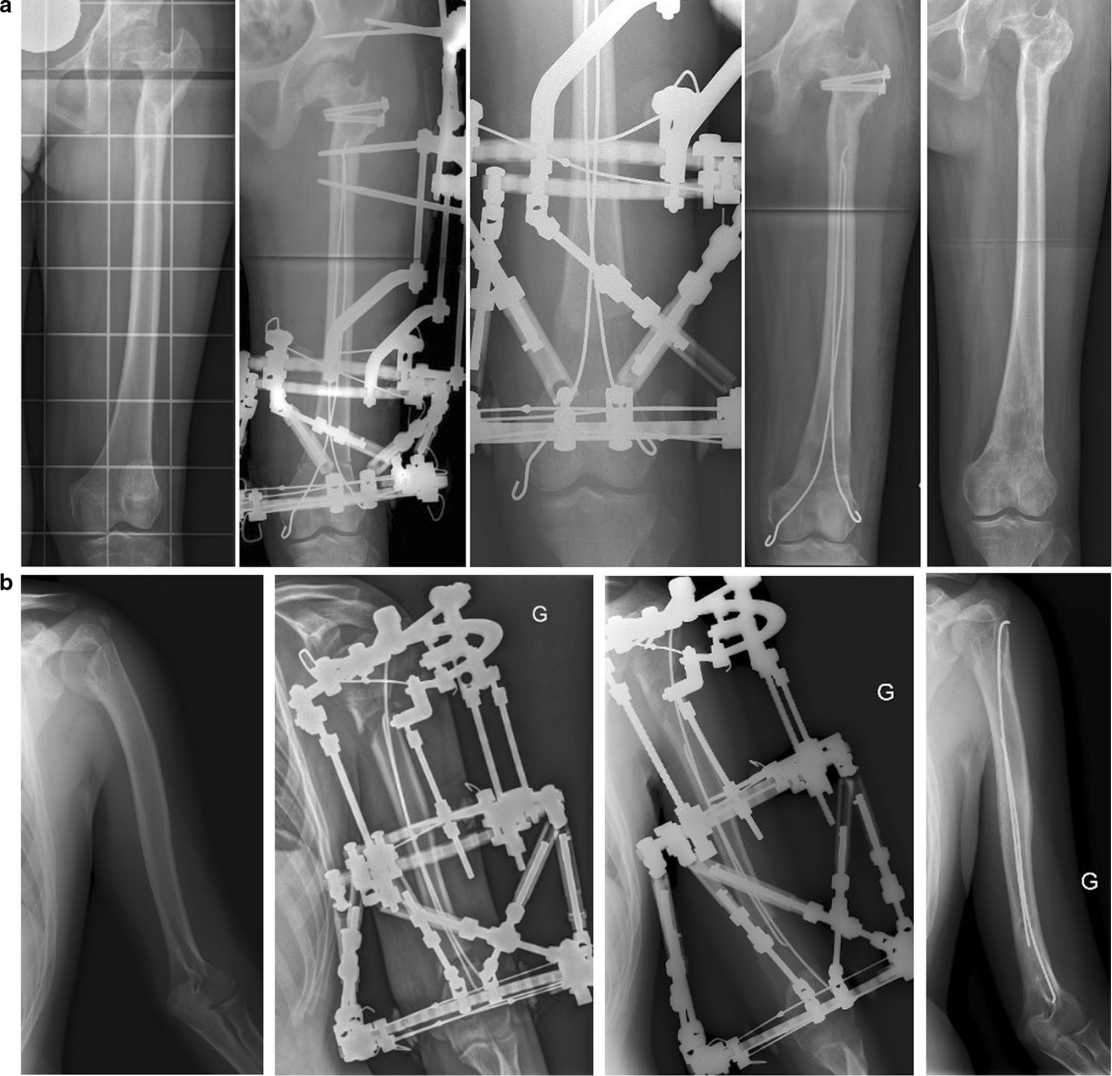
Examples of lengthening using combined technique: **a** femur lengthening and simultaneous valgus deformity correction; **b** bifocal humeral lengthening with bipolar sliding FIN and TSF^®^

If limb lengthening is associated with a deformity correction, the surgeon can align the concave curves of both nails toward the convexity of the deformity. In doing so, the progressive realignment is effective and the stability of the fragments is increased. The elasticity of both nails allows for simultaneous lengthening and a gradual correction of angular deformity (Fig. 4a).

Bipolar, sliding FIN is more appropriate in cases requiring bifocal lengthening. For instance, in the humerus in Fig. 4b, an antegrade nail has been inserted through the proximal metaphysis, and a distal retrograde nail has been inserted using the distal lateral supra-condylar approach. During the distraction period, the nails slide in opposite directions. So, after frame removal, both the proximal and distal regenerated areas are protected by at least one curved nail. Its alignment averts a secondary angular deformity.

In cases of forearm lengthening, we use one nail for each bone. The nails have to be aligned towards each other, making two opposed curves. The retrograde radial nail is always inserted through an incision over the distal metaphysis, and the antegrade ulnar nail is always inserted using a posterolateral olecranon approach. The advantage of FIN in resisting lateral displacement is made evident when lengthening small diameter bones like those in the forearm.

**Fig. 5:**
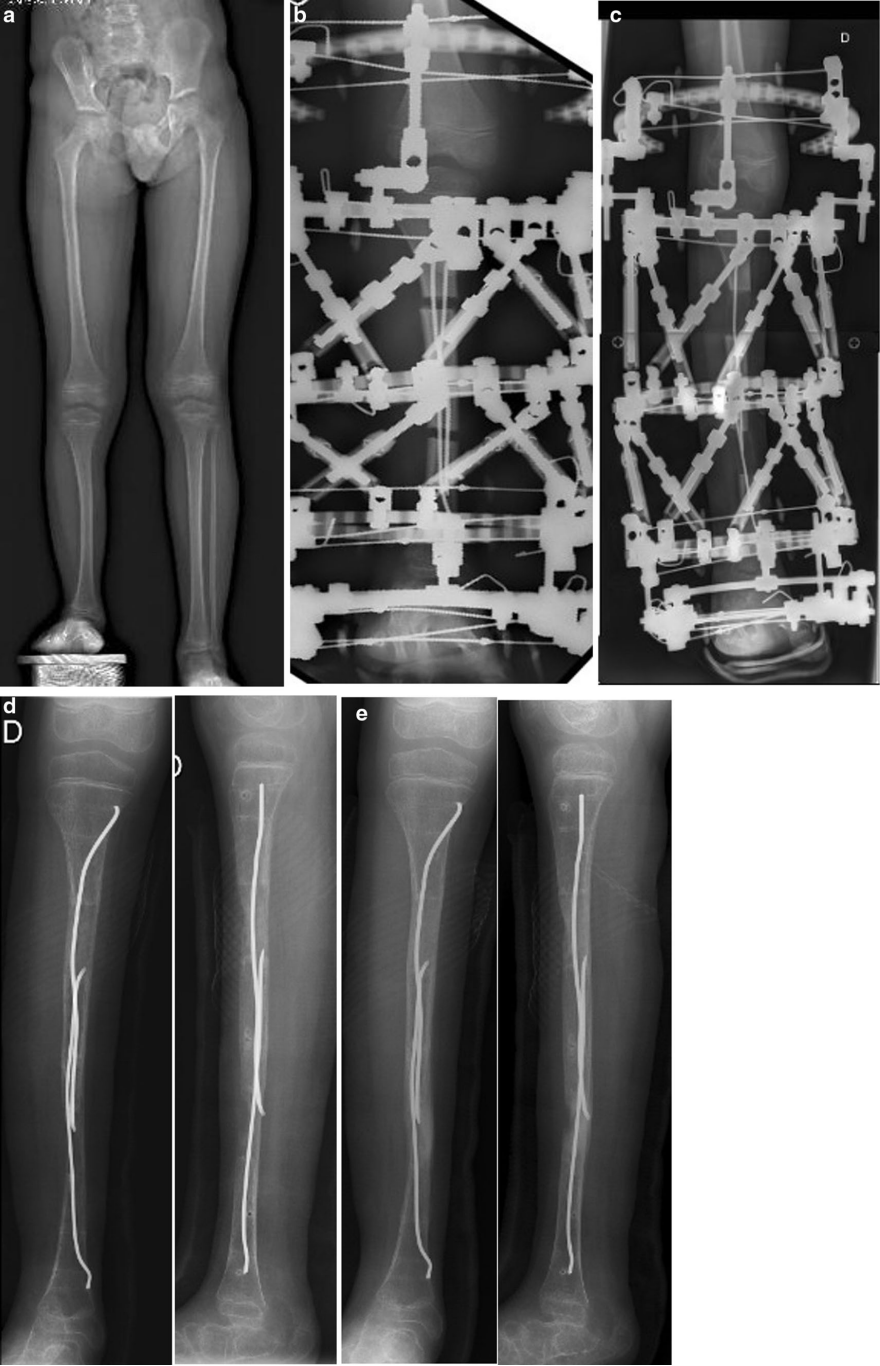
Bipolar tibia lengthening in patient with fibular aplasia: **a** X-ray image of lower limbs before treatment; **b** day 10 of distraction period; **c** day 46 of distraction period (end of lengthening); **d** day of frame removal, distal regenerated bone is weak, without anterior cortex, but protected by intramedullary nail; **e** 6 weeks after frame removal, distal regenerated area is solid, with thickened cortices and no angular deformity

When FIN is left in situ after frame removal, it also protects the weak regenerated area until bone consolidation is complete (Fig. 5). Usually, FIN should be removed within 6–9 months after frame removal, once the range of initial motion of adjacent joints is restored.

### Combined technique using FIN in limb lengthening for congenital or acquired (growth arrest following trauma or neonatal osteomyelitis) upper- and lower-limb length discrepancy [29]

Using a prospective study, we compared the HI of two groups of children who had undergone upper- and lower-limb lengthening carried out using the Ilizarov external fixator alone (194 cases) or a combination of the Ilizarov fixator and FIN (92 cases). The HI was lower in the combined technique group. Significant differences were noted in:

- congenital pathologies: monofocal, monosegmental lengthening of the femur and forearm, bifocal lengthening of the tibia, and polysegmental lengthening;
- acquired discrepancies: monofocal tibia, bifocal femoral, and forearm lengthening.

The HI difference between the two techniques varied from 2 to 19.1 days/cm. This means that patients in the combined technique group using FIN required 20–33% fewer days in the external fixator than those in the conventional Ilizarov technique group. The largest difference in HI was noted in cases of bifocal acquired femoral discrepancies (59.9%) and monofocal acquired forearm discrepancies (51.3%). The association of the Ilizarov device and FIN showed a mean HI reduction of 7 days/cm. Furthermore, the Ilizarov plus FIN technique avoided and/or considerably decreased the number of complications related to longer-term external fixation, such as pin-tract infection, osteomyelitis, and fractures and deformities after frame removal. However, there were eight cases of skin irritation where the bent FIN exited the bone; these required one nail to be removed per bone—two during the distraction period and six during the consolidation phase. The removal of those nails had no influence on the final outcomes.

The combined, simultaneous Ilizarov external fixator and FIN technique (with two oppositely curving nails) has been described positively by other authors. They have shown the advantages of shorter durations of external fixation, good protection of the lengthened bones against refracture, earlier rehabilitation and its applicability to children [14, 30–32]. Different published studies have used monolateral [9, 14, 30] and circular [14, 31, 32] external fixators. All these authors agreed that the key element of the combined technique was FIN.

### FIN in limb lengthening and deformity correction in patients with Ollier disease [33]

Dyschondroplasia, or Ollier disease, is a rare, non-hereditary skeletal disorder. This disease is responsible for various troubles linked to the development of multiple enchondromas secondary to growth disturbances: limb-length discrepancy, complex deformities and pathological fractures. These orthopaedic complications require more specific management.

A retrospective study [33] assessed the efficiency of FIN combined with a circular external fixator (such as the Ilizarov or Taylor Spatial Frame^®^) for lower-limb lengthening and associated deformity correction procedures, against the results achieved with external fixation alone, i.e. without FIN. The mean HI was significantly lower in patients who underwent lengthening and deformity correction using the combined technique. The HI varied from 19 to 28.2 days/cm for monosegmental lengthening, and from 10.9 to 12.3 days/cm for polysegmental lengthening. The average duration of external fixation treatment was thus reduced by about 8 days for each centimetre of monosegmental lengthening.

In the group treated using the conventional Ilizarov technique, three pathological fractures at the site of enchondromas and three deformities at the lengthening site were observed after the external fixator’s removal from 37 patients. On the other hand, among the seven patients treated using the combined technique, no secondary fractures were observed over the 25-month follow-up.

### Bioactive FIN in lower-limb deformity correction in children with X-linked hypophosphatemic rickets (XHPR) [34]

Surgical procedures to correct multiplanar bone deformities may be indicated for the prevention of secondary orthopaedic complications in children with XHPR, however, different problems related to these procedures have been reported: increased rates of delayed union, recurrent deformity, deep intramedullary infection, refracture and pin-tract infection.

In a retrospective study, we compared the results of corrections in children with XHPR who had undergone treatment using either an Ilizarov device alone or a combined technique (Ilizarov fixator plus bioactive hydroxyapatite-coated FIN). The results of surgery were compared in short-term (2–6 months) and long-term (over 5 years) follow-up.

Applying the combined technique demonstrated a considerably lower duration of external fixation (87.4 days) than the external fixator technique alone (124.7 days), as well as a decrease in the number of infectious complications, an absence of secondary fragment displacement during correction, and no deformity at the osteotomy after frame removal. In most cases, at long-term follow-up, neither recurrence nor development of any deformity was observed. In children treated using the combined technique, no recurrent deformity appeared around the FIN left in situ. Nevertheless, some new deformities appeared, either in the distal femoral or proximal tibial metaphysis during residual spontaneous growth. These areas were no longer protected by the intramedullary nails. The bioactive layers prevented the migration of the nails at total weight-bearing and long-term follow-up.

We suggest the use of intramedullary nails coated with hydroxyapatite or another bioactive layer in patients with metabolic bone disorders and in cases where bone consolidation is compromised [33–35].

### Disadvantages of the combined technique for limb lengthening and deformity correction [9, 14, 25, 29–36]

**Fig. 6:**
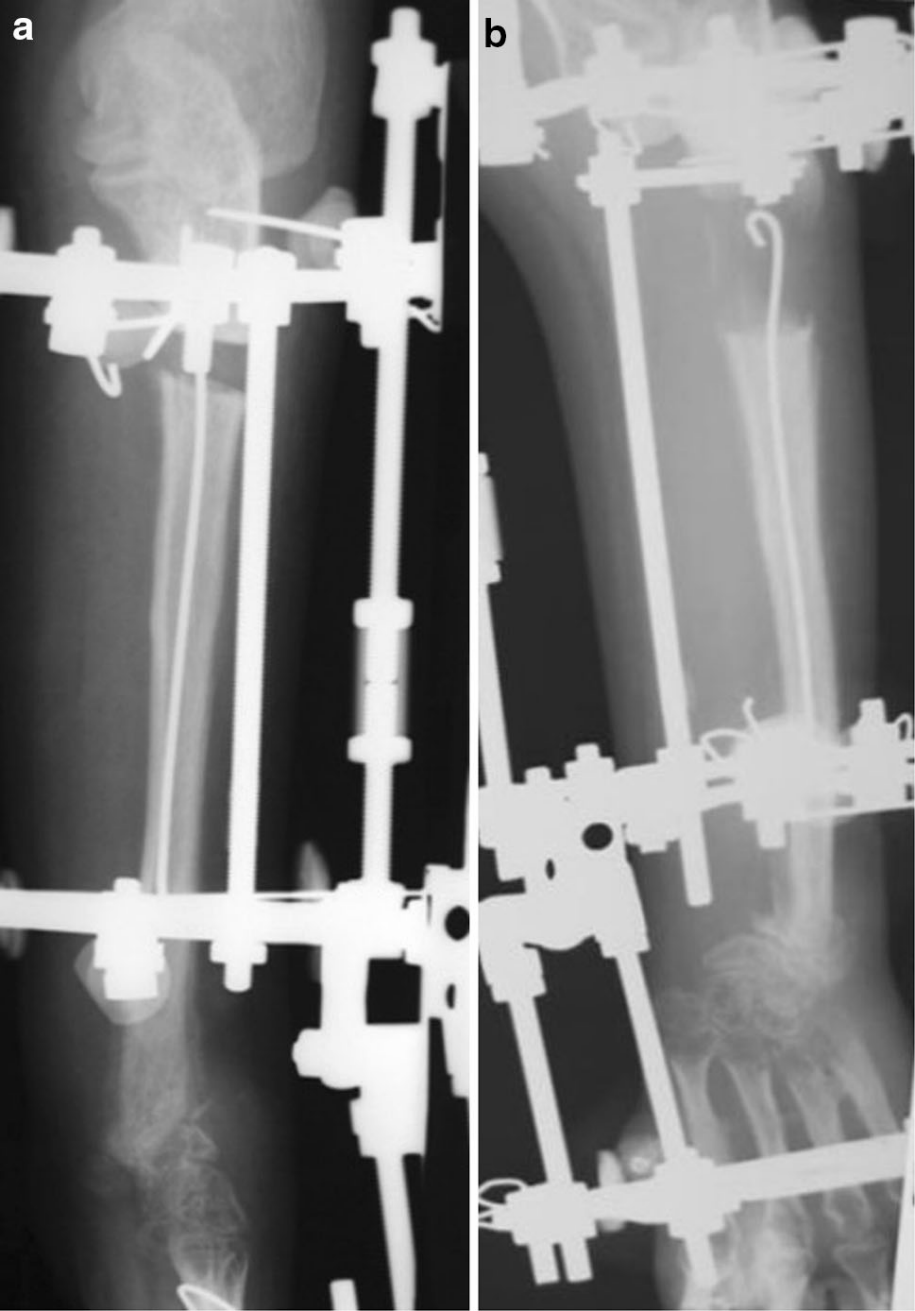
Example of internal migration of a nail into the medullary canal due to its insertion through the osteotomy site: **a** beginning of lengthening; **b** migration of nail into medullary canal during distraction period of treatment
  premature consolidation due to the stimulation of the regenerated bone area induced by the nail progressively sliding into the medullary canal (Fig. 7);

Here, we list some opinions and judgments about the potential inconveniences of using the combined technique rather than the Ilizarov procedure alone, as well as complications which have been observed in different groups of our patients:

1. The surgical procedure lasts 10–20 min longer.
2. In patients with congenital or acquired limb-length discrepancies, nail removal should be adequately planned. This usually requires a period of outpatient hospitalisation.
3. Besides the general complications of surgery and anaesthesia, there are complications specifically related to the insertion or removal of FIN:
  A prominent nail end, leading to irritation or perforation of the skin, due to insufficient trimming or external migration of the nail;
  nerves or tendons rubbing the prominent end of a nail;
  Internal migration of nails in the medullary canal, due either to their insertion through the osteotomy (Fig. 6) or insufficient bending of their trailing ends;

**Fig. 7:**
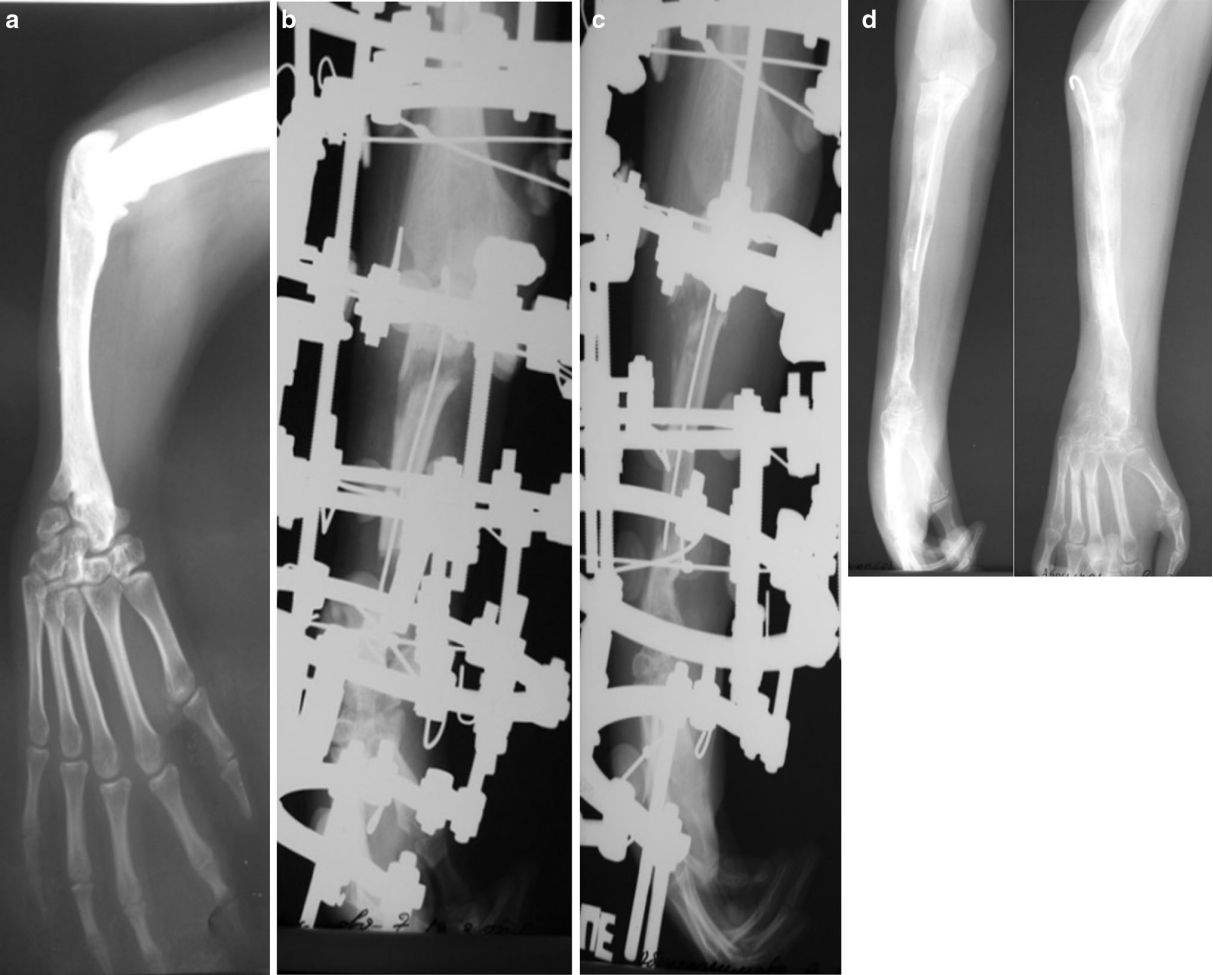
Example of forearm lengthening: **a** X-ray image of forearm before treatment; **b** premature bone consolidation noticed; **c** distraction is restarted after an unscheduled osteotomy; **d** X-ray image after frame removal with planned gain in length achieved
  intramedullary nails become blocked against the wires or half-pins of the external fixator;
  difficulty in maintaining an appropriate rate of distraction, when only one of the two forearm bones is fixed with a nail;
  the external fixator is removed before the adequate bone consolidation—is essential to understand that FIN is not a stand-alone osteosynthesis procedure and it cannot ensure that there will be no secondary fractures or deformities when the quality of the regenerated bone is insufficient to allow the removal of the external fixator;
  osteomyelitis related to FIN.
4. It may take surgeons some time to master the difficult technique of FIN using two nails.

## Conclusion

Flexible intramedullary nailing (FIN) is a minimally invasive procedure which provides multiple advantages during limb lengthening and deformity correction using an external circular fixator. When correctly performed, FIN respects bone biology, which is mandatory for good healing.

The advantages of the combined technique (circular fixator plus FIN) for bone lengthening are a lower HI, a shorter distraction-consolidation time, the reduced rate of septic and bone complications, the ability to correct deformities gradually, the increased stability of bone fragments (no secondary translation during the lengthening) and, finally, the biomechanical benefits to the regenerated bone of the transition from partial to full weight-bearing during walking.

Using flexible intramedullary nails with a bioactive (e.g. hydroxyapatite) coating seems a promising treatment for complex limb deformities in metabolic bone disorders and in cases where bone consolidation is compromised.
